# Role of glycosphingolipid biosynthesis coregulators in malignant progression of thymoma

**DOI:** 10.7150/ijbs.83468

**Published:** 2023-08-21

**Authors:** Xin Zhang, Bo Zeng, Haoshuai Zhu, Rui Ma, Ping Yuan, Zhenguang Chen, Chunhua Su, Zhihao Liu, Xiaojing Yao, Aurora Lawrence, Zhenguo Liu, Jianyong Zou

**Affiliations:** 1 Department of Thoracic Surgery, the First Affiliated Hospital of Sun Yat-Sen University, Guangzhou, 510080, China.; 2 Guangdong Provincial Key Laboratory of Colorectal and Pelvic Floor Disease, The Sixth Affiliated Hospital of Sun Yat-sen University, Guangzhou, 510655, China.; 3 School of Medicine, Stanford University, 450 Serra Mall, Stanford, CA 94305, USA.

**Keywords:** Mass spectrometry analysis, Thymoma, Metabolic reprogramming, Glycosphingolipid

## Abstract

As the most common malignancy from mediastinum, the metabolic reprogramming of thymoma is important in its development. Nevertheless, the connection between the metabolic map and thymoma development is yet to be discovered. Thymoma was categorized into three subcategories by unsupervised clustering of molecular markers for metabolic pathway presentation in the TCGA dataset. Different genes and functions enriched were demonstrated through the utilization of metabolic Gene Ontology (GO) analysis. To identify the main contributors in the development of thymic malignancy, we utilized Gene Set Enrichment Analysis (GSEA), Gene Set Variation Analysis (GSVA), and Kyoto Encyclopedia of Genes and Genomes (KEGG) pathway enrichment analysis. The prognosis of thymoma was evaluated by screening the essential pathways and genes using GSVA scores and machine learning classifiers. Furthermore, we integrated the transcriptomics findings with spectrum metabolomics investigation, detected through LC-MS/MS, in order to establish the essential controller network of metabolic reprogramming during thymoma progression. The thymoma prognosis is related to glycosphingolipid biosynthesis-lacto and neolacto series pathway, of what high B3GNT5 indicate poor survival. The investigation revealed that glycosphingolipid charts have a significant impact on metabolic dysfunction and could potentially serve as crucial targets in the clinical advancement of metabolic therapy.

## Introduction

The most common primary mass in the anterior mediastinum is thymoma, which originates from the thymic epithelium. At present, the molecular mechanisms underlying thymoma are still not understood. Multidisciplinary approach is necessary for treating thymoma, with surgery being the primary method for achieving cure 1. However, up to 30% to 50% of patients may develop advanced, recurrent, metastatic, or refractory tumors. Moreover, patients experiencing advanced stages of thymoma typically exhibit unsatisfactory treatment outcomes 2, 3. Hence, it is crucial to discover dependable predictive biomarkers for the identification of thymoma patients, as this would enhance the chances of survival.

Carcinogenesis often involves the reprogramming of metabolism, which has significant effects on the tumor microenvironment, cellular differentiation, and the expression of genes. The alteration of metabolism has been demonstrated to impact the growth, proliferation, and invasion of tumor cells in various manners. For instance, it can fulfill the energy requirements of rapidly dividing cancer cells and modify biological processes by influencing the makeup of substances 4. The identification of metabolically significant biomarkers aids in the identification of abnormal organism-specific alterations, and there is an increasing body of evidence connecting metabolic irregularities to unfavorable prognosis in various cancers, including clear cell renal cell carcinoma, colorectal cancer, and endometrial cancer 5-7. Nevertheless, the precise expression patterns of genes associated with metabolism in thymoma remain uncertain.

The beta-1,3-N-acetylglucosaminyltransferase (B3GNT) family consists of a collection of enzymes that demonstrate an extraordinary capacity to facilitate the transfer of N-acetylglucosamine (GlcNAc) from UDP-GlcNAc to specific target molecules 8. The formation of beta-1,3-linked GlcNAc residues through this enzymatic process has significant implications in diverse biological scenarios. Currently, the B3GNT family comprises eight known members, namely B3GNT1 to B3GNT8, each displaying unique characteristics and functions. Notably, the disruption of the B3GNT group has been identified as a crucial element in the advancement and advancement of cancer 9. For example, abnormal levels of B3GNT1 expression have consistently been detected in different types of cancer, appearing as an increase in its occurrence. The elevated expression is strongly linked to negative consequences like accelerated tumor growth, enhanced ability to spread, and worse prognosis 10. Moreover, within the B3GNT family, B3GNT5 has garnered considerable attention due to its strong association with glioma progression 9.

The ST3GAL family is composed of sialyltransferases, which are enzymes categorized under the sialyltransferase 3 (ST3) subfamily 11. Six members of this family has been reported. Glycosyltransferases known as sialyltransferases facilitate the transfer of sialic acid residues from donor molecule, typically CMP-sialic acid, to acceptor molecules. This process leads to the incorporation of sialic acid and glycan structures. The members of this family paly an important role in different biological processes, like cell communication, growth, immune defense, and progression of diseases. Changes in the expression or function of ST3GAL family members have been detected in various conditions, such as malignancy, inflammation, and neurodegenerative ailments 12.

For this study, we utilized mRNA expression information of individuals diagnosed with thymoma from The Cancer Genome Atlas (TCGA) repository to construct a metabolic prognostic signature. Extensive bioinformatics analysis revealed substantial alterations in the metabolomics of thymoma. Afterwards, thymoma metabolites and metabolic pathways were investigated using differential metabolomics. Afterwards, a correlation analysis was conducted to examine the relationship between distinct metabolic pathways and significant clinical phenotypes and we verified the results with the clinical samples and data. In conclusion, we have discovered the most predictive metabolic pathways and crucial genes in thymoma. These findings could offer fresh insights into examining the prognosis of thymoma and formulating personalized metabolic treatment strategies.

## Methods

### Patients and tissue samples

All the thymoma tissues and adjacent normal thymic tissues were collected from patients who underwent surgery in thoracic surgery, the First Affiliated Hospital, Sun Yat-sen University. All patients have not received any therapy before surgery. The samples were collected immediately after removing the resected thymoma, and stored at -80 °C. All the participating patients have signed informed consent form and the study was approved by the Ethics Committee of the First Affiliated Hospital, Sun Yat-sen University.

### Thymoma molecular subtyping and survival analysis

Downloaded from the UCSC Xena database (https://xenabrowser.net/datapages/), the RNA-seq data for 121 TCGA-thymoma (THYM) samples was obtained in log2 (count+1) units. The phenotypes of the THYM samples were also downloaded from GDC Hub. The analysis of cancer molecular subtypes was conducted using the algorithms 'CancerSubtypes' and 'ClassDiscovery'^13^. Cluster analysis was performed using the RNA expression matrix and average linkage method, employing the 'SNFCC' approach.

Following subcategorization, the patients with distinct thymoma subtypes underwent survival analysis utilizing the survival and survminer software packages 14. Using the top-notch Separation algorithm to distinguish between high and low expression, the samples were automatically classified based on variations in survival rates. Each group consisted of a minimum of 10% of the total samples 15.

### Analysis of Prognostic differentially expressed genes and functional enrichment

An analysis of differential gene expression was conducted on the samples from two clusters that exhibited the most notable disparity in survival. To normalize gene expression profiles and remove low-expression genes, the DESeq algorithm was employed 16. To choose differentially expressed genes, the requirements included a log2-fold change ≥1.5 and a Benjamini-Hochberg (B-H) adjusted *P value* <0.05. The differentially expressed genes were clustered based on their expression patterns using unsupervised cluster analysis. Subsequently, the clusters underwent Gene Ontology (GO) enrichment analysis to investigate their potential biological roles using ToppGene Suite (https://toppgene.cchmc.org) 17.

### Gene set enrichment analysis and gene set variation analysis

Based on the algorithms 'clusterProfiler' and 'AnnotationHub'^18^, ^19^, we performed a gene-set enrichment analysis (GSEA, http://software.broadinstitute.org/gsea/index.jsp) on thymoma tissues from the TCGA-THYM project. The minimum gene set size parameter for the algorithm was set to 10, while the maximum gene set size was 500. Significantly enriched pathways were determined by considering terms with a permutation test number of 1000 and a B-H corrected P value less than 0.05.

Furthermore, GSVA is a nonparametric unsupervised technique utilized for assessing the outcomes of gene set enrichment in microarray or RNA-seq data 20. The gene list of central pathways was gathered by incorporating the KEGG (http //www.genome.ad.jp/kegg/), REACTOME (https //reactome.org/) and PathCards databases (http //pathcards.genecards.org/). For normalized scoring of gene sets per cell, the GSVA and GSEABase packages were used 21. To assess the lasting impact on patients, we conducted survival analysis on pathways scored using GSVA 22.

### Analysis of differential metabolic pathways

GSVA is an unsupervised and nonparametric approach that assesses the enrichment of gene sets in the transcriptome. Identifying the metabolic pathways enriched in a sample can be achieved by converting the gene expression matrix into a gene set expression matrix. The project utilized GSVA to assess the variations in gene sets among different metabolic pathways in sample clusters 23. By employing the approach described by Wu et al., we successfully detected gene clusters associated with 46 metabolic pathways in tumors. These pathways were standardized and quantified using GSVA algorithms. The differential analysis of metabolic pathway GSVA scores in sample clusters was performed using the B-H algorithm from the Limma R package, resulting in the generation of a bar chart 24.

### Significance of differential metabolic pathways in thymoma classification

First, the GSVA scores of differential metabolic pathways were extracted and matched with the classification of clusters. We built a machine learning classifier for the two groups of patients with thymoma who had the most distinct survival rates, utilizing linear regression, decision tree, and random forest analyses. By employing recursive feature selection, the decision tree algorithm categorizes the training dataset and produces a tree composed of nodes and directed edges 25. The nodes are divided into internal and leaf nodes, with an internal node representing a feature and a leaf node representing a class. A random forest is a type of classifier that consists of multiple decision trees, and the final class prediction is based on the most frequently occurring class among these trees. The random forest method is highly valuable for error balancing and accuracy maintenance 26. It can be applied to handle datasets with multiple variables, generate classifiers with high accuracy, and evaluate feature importance using decision trees. Furthermore, we assessed the precision, recall, and AUC curve to determine the accuracy and stability of the machine learning model. Additionally, we built a SHAP model and computed the SHAP score to evaluate the impact of key attributes on the categorization of two groups of thymoma patients with the most significant contrast in survival 27.

### Mass spectrometry-based metabolomic profiling

We picked 10 specimens, comprising of five samples of thymoma tissue and five samples of para-tumor tissue. The samples were analyzed utilizing ultra-performance liquid chromatography (UPLC, ExionLC AD, USA) and tandem mass spectrometry (MS/MS; QTRAP®, USA). Various statistical techniques were employed to examine variations in metabolites among the samples 28.

The mass spectrometry data was processed using Analyst 1.6.3.A combination of the sample extracts 29 was used for quality assurance. To ensure the consistency of measurement under identical operating conditions, analytical samples were periodically supplemented with quality control samples, with a frequency of one every ten analytical samples. The total ion current (TIC) chromatogram and multiple reaction monitoring (MRM) multipeak chromatogram were obtained. To identify the distinctive ion of every compound, a triple quadrupole mass spectrometer was employed for screening. The detector captured the signal intensity (counts per second) of the characteristic ion. The samples' mass spectrometer output files were utilized for chromatographic peak integration and calibration using MultiQuant software 30. The chromatographic peak area indicates the proportionate amount of the corresponding compound. All data related to the integration of chromatographic peak areas were exported and saved. In order to ensure the accuracy of qualification and quantification, we adjusted the chromatographic peak of each detected metabolite in various samples by considering the metabolite's retention time and peak type, allowing for comparison of their levels 31.

### Orthogonal partial least squares-discriminant analysis

By utilizing the metabolomic data obtained earlier, we have the capability to conduct metabolite identification and analyze the quality of sample data. We choose differential metabolites and perform predictive and analytical functions on the metabolites present in the samples. OPLS-DA (Orthogonal Partial Least Squares-Discriminant Analysis) combines orthogonal signal correction and PLS-DA to separate the matrix data of independent variables into two components: irrelevant information and dependent variable-related information. By utilizing this approach, it becomes possible to maximize variations between different groups, which aids in the detection of distinct metabolites 32. Additionally, it helps eliminate irrelevant disparities in order to pinpoint specific variables that differ significantly. Ultimately, this method enhances the outcomes of differential analysis. The OPLS-DA model utilized Variable Importance in Projection (VIP) to initially identify distinct metabolites among groups 33. Further differential metabolite selection was based on the P value or fold change obtained from univariate analysis. Differential metabolites were determined by setting a threshold of VIP ≥ 1.0 and fold change ≥ 2 or fold change ≤ 0.5. In other words, if the ratio of a metabolite in the tumor group to that in the control group was ≥ 2 or ≤ 0.5, it was considered to have statistically significant difference.

### Functional annotation and enrichment analysis of differential metabolites

Using the Kyoto Encyclopedia of Genes and Genomes (KEGG) database, we conducted an analysis on the connections and routes of distinct metabolites in living beings. This primarily encompassed the potential metabolic pathways of carbohydrates, nucleotides, and amino acids, as well as the degradation of organic substances. Additionally, we performed a thorough annotation of enzymes for a range of reactions.* P* value < 0.05 was considered statistically significant 34.

The conventional analysis of enrichment, which relies on the hypergeometric distribution, is primarily suitable for identifying metabolites that have significant upregulation or downregulation. However, it may overlook metabolites that lack significant differential expression despite their crucial biological importance. Metabolic set enrichment analysis (MSEA) converts metabolomic data into a range of predetermined metabolic sets without specifying the threshold for differential metabolites in advance 35. Metabolic sets with significant differences were identified using MSEA.* P* value < 0.05 for pathway enrichment was considered statistically significant.

### Effects of the core pathway on survival and identification of target genes

The machine learning classifier mentioned earlier identified the regulatory metabolic pathway of utmost significance. Additionally, we acquired the GSVA scores for this pathway, which were automatically partitioned using the survival and survminer packages to derive an optimal threshold value. We examined the impact of this pathway on long-term survival, as determined by DSS, PFI, and OS. Additionally, we acquired the gene expression pattern of this central pathway and investigated gene correlation through Pearson correlation analysis. The gene expression differences corrected by the B-H algorithm in Limma were used to determine the differential expression of these genes. Finally, genes with Log|FC| ≥1.0 and an adjusted *p value* ≤ 0.05 were selected as targets. Continuous variables may have nonlinear effects on survival. In order to eliminate the non-linear impacts of expression profiles, the smoothHR and Hmisc algorithms were employed to construct a survival prediction model that links differential target gene expression with overall survival (OS). Additionally, the risk ratio was calculated for each level of continuous gene expression compared to the baseline, providing further evaluation of the effects of genes on long-term survival. Afterwards, the validation of the effects of gene expression on the long-term survival (DSS, PFI, and OS) in patients with thymoma was conducted by utilizing the survival and survminer packages.

### Immunohistochemistry (IHC) and Kaplan-Meier *survival* analysis

IHC staining for B3GNT5 and ST3GAL6 was performed on all 28 thymoma and adjacent normal tissue samples, which were fixed in 10% neutral buffered formalin, paraffin-processed and embedded. The deparaffinized tissue sections (4 mm thick) were stained with antibody against B3GNT5 and ST3GAL6 (Santa Cruz, Dallas, Texas, USA) for immunohistochemical analysis. Images of immunohistochemistry staining were photographed under a light microscope (Leica, Wetzlar, Germany). IHC scores were independently evaluated by two individuals. The final score was the multiplication of the positive ratio value (0-3) and the intensity value (0-3) of the immunoreactive cells. The scores ranging from 0-4 were defined as low expression, while the scores > 4 were considered high expression. Kaplan-Meier *survival* analysis was conducted by GraphPad Prism version 9.5.1.

## Results

### Thymoma subtype identification and differential analysis

**Figure 1:**
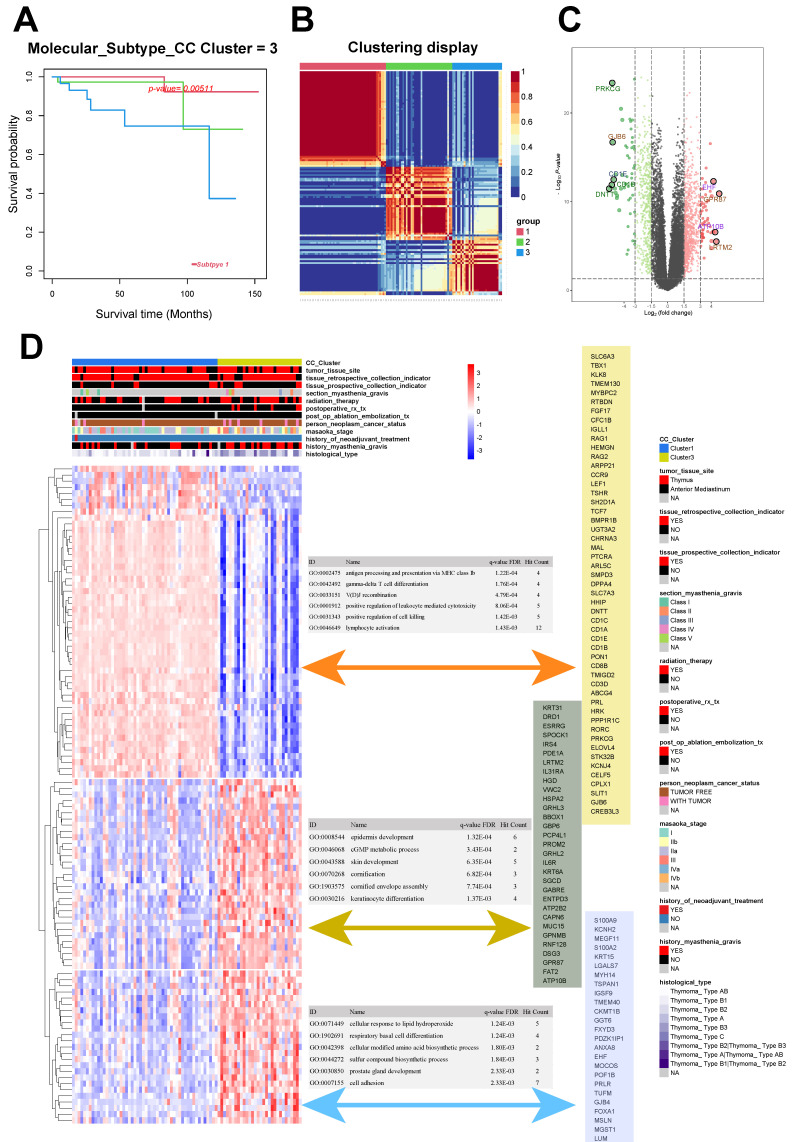
Consensus clustering and different metabolic profiles between the two clusters. **A.** Survival analysis of thymoma subgroups. **B** Consensus matrix heatmap analysis. **C** Volcano plot of differentially expressed genes between subgroup 1 and 3. **D** Correlation analysis and GO enrichment analysis between differentially expressed genes and clinical phenotypes.

The 121 samples of THYM were categorized into three distinct groups: Group 1 (52 cases), Group 2 (39 cases), and Group 3 (30 cases). The chi-squared test resulted in a *Q* value of 10.6 and a* P* value of 0.005 for the groups (Fig. 1A-B). The unsupervised heat map of tissue gene expression data indicated that this classification was trustworthy, with groups showing relative autonomy and strong correlation within each group (Fig. 1B). Groups 1 and 3 exhibited the most notable variation in survival rates, defined as the good-outcome and poor-outcome groups correspondingly (Fig. 1A).

The analysis of differential gene expression, using the transcription data of Groups 1 and 3, revealed 1370 differentially expressed gene. Among these genes, 524 were found to be downregulated while 846 were upregulated (Fig. 1C). We gathered the clinical data related to the samples and conducted an unsupervised cluster analysis on the expression levels of the top 100 genes that showed differential expression. The findings indicated that the genes exhibiting differential expression in Groups 1 and 3 could potentially be linked to the histological classification of the tumor and past occurrence of myasthenia gravis (Fig. 1D).

After clustering, the genes that showed differential expression were categorized into three distinct clusters. Cluster 1 was mainly associated with GO:0002475 (antigen processing and presentation via MHC class Ib; *P* < 0.001), GO:0042492 (gamma-delta T-cell differentiation; *P* < 0.001), and GO:0033151 (V[D]J recombination; *P* < 0.001). Cluster 2 was mainly associated with GO:0008544 (epidermis development; *P* < 0.001), GO:0046068 (cGMP metabolic process; *P* <0.001), and GO:0043588 (skin development; *P* < 0.001). Cluster 3 was mainly associated with GO:0071449 (cellular response to lipid hydroperoxide; *P* = 0.001), GO:1902691 (respiratory basal cell differentiation; *P* = 0.001), and GO:0042398 (cellular modified amino acid biosynthetic process; *P* = 0.002; Fig. 1D).

### Construction of a pathway interaction network of different subtypes and survival analysis

**Figure 2:**
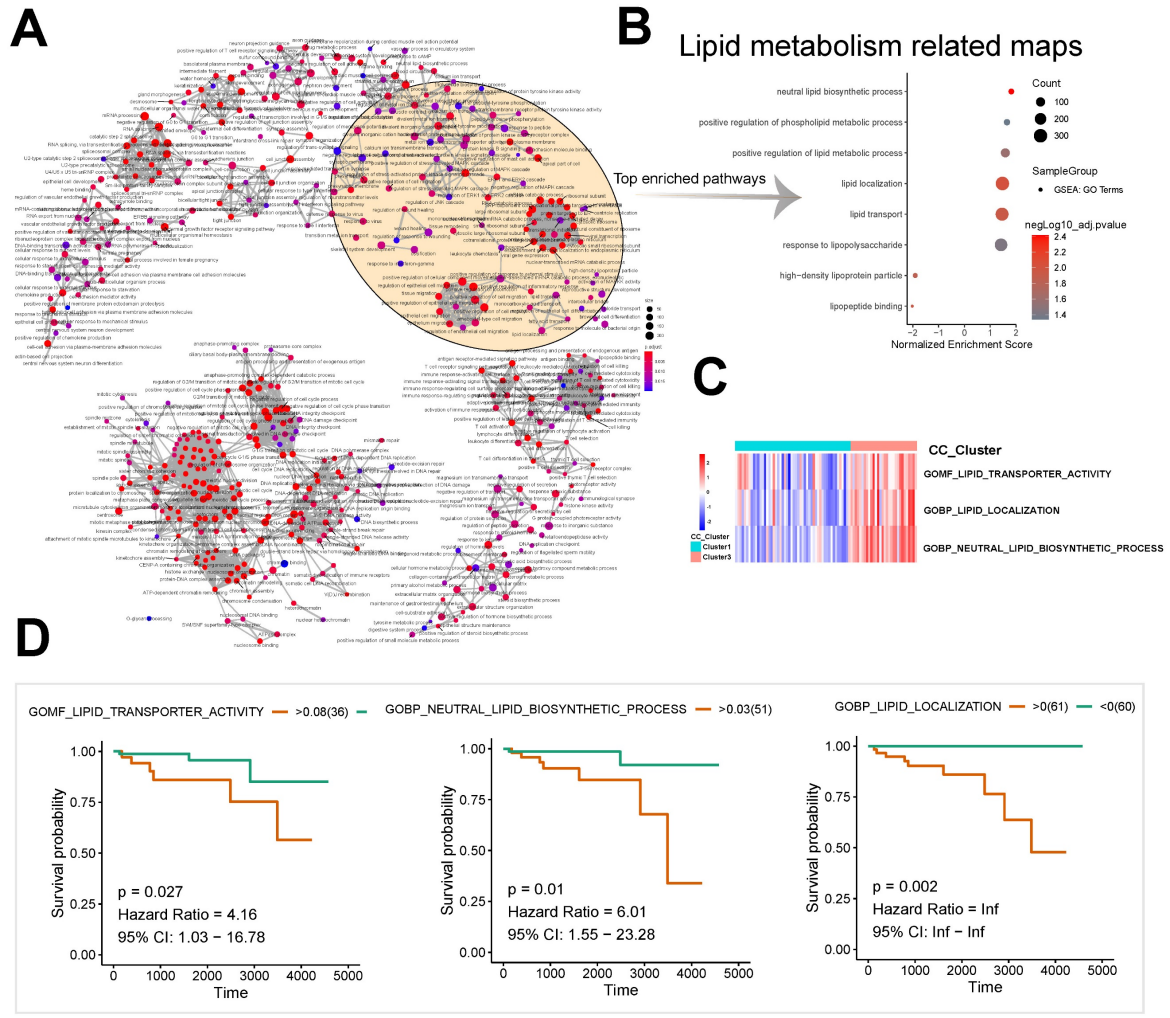
Construction of the pathway interaction network of different subtypes and survival analysis. **A, B** GSEA enrichment analysis of differentially expressed genes. **C** GSVA analysis of differentially expressed genes. **D** The lipid metabolism related pathway survival analysis based on GSVA score.

A pathway interaction network was derived from the enriched differentially expressed genes of subtypes 1 and 3 by GSEA (Fig. 2A). Lipid metabolism related maps and immune/inflammation related maps were significantly enriched (Fig. 2B). Significant enrichment was observed in neutral lipid biosynthetic process (n = 32, normalized enrichment score [NES] = 1.82, P < 0.001), positive regulation of phospholipid metabolic process (n = 44, NES = 1.65, P = 0.004), and positive regulation of lipid metabolic process (n = 121, NES = 1.59, P = 0.002) among pathways related to lipid metabolism (Fig. 2B).

The unsupervised cluster heatmap based on the GSVA score showed significant differences between Groups 1 and 3 in the components of the following pathways: GOMF: lipid transporter activity; GOBP: lipid localization; and GOBP: neutral lipid biosynthetic process (Fig. 2C). The results suggested important roles of lipid metabolism in the proliferation and differentiation of the two subtypes of thymomas. Based on the GSVA scores of pathways, we also found that the long-term survival of patients with thymomas was closely associated with GOMF: lipid transporter activity (*P* = 0.027, hazard ratio [HR] = 4.16, 95% confidence interval [CI]: 1.03-16.78), GOBP: lipid localization (*P* = 0.002, HR = Inf, 95% CI: Inf-Inf), and GOBP: neutral lipid biosynthetic process (*P* = 0.01, HR = 6.01, 95% CI: 1.55-23.28) (Fig. 2D).

### Analysis of differential metabolic pathways

**Figure 3:**
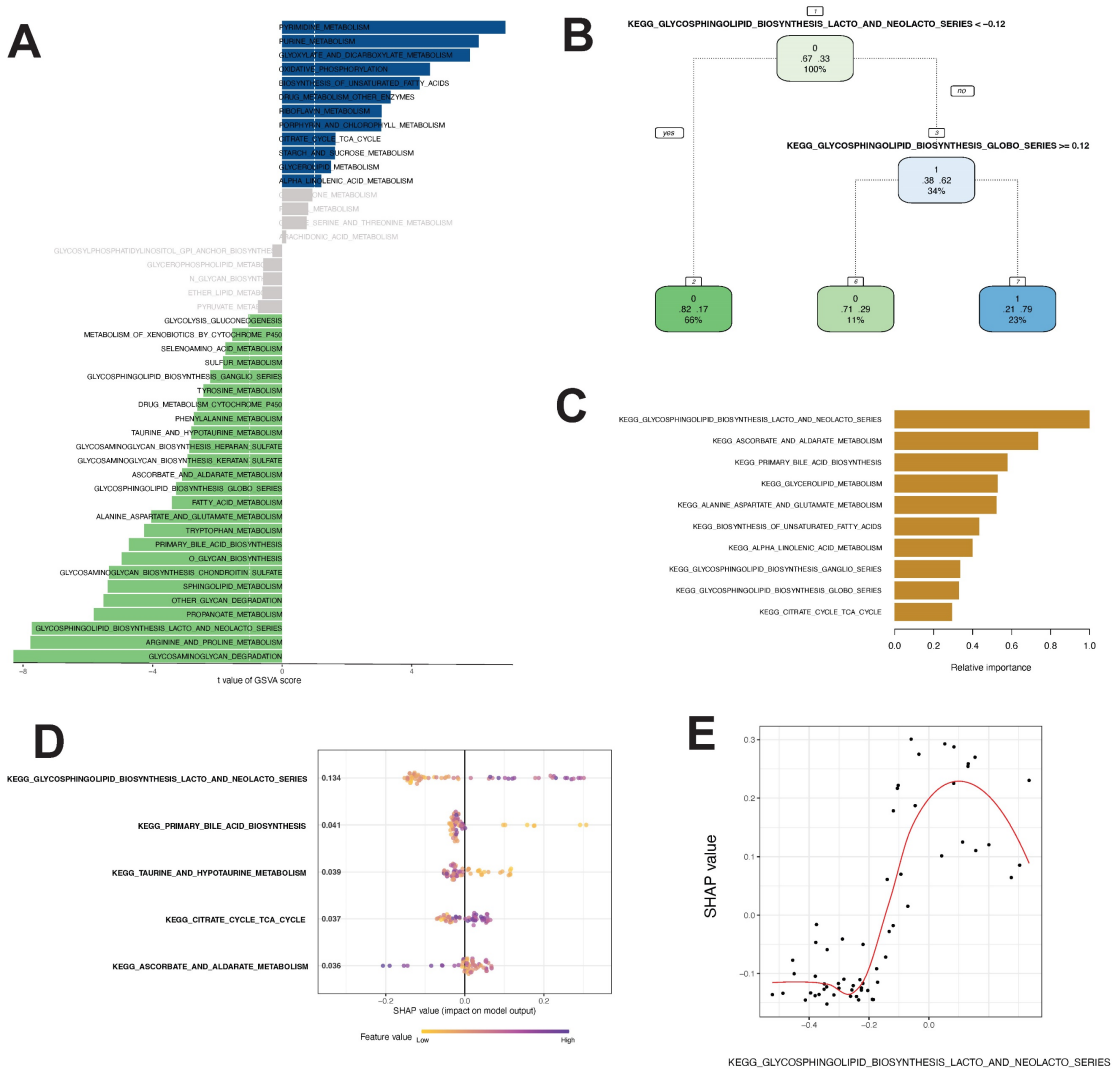
Differential metabolic pathway analysis and core pathway construction in thymoma. **A** Twenty-five differential metabolic pathways were identified via GSVA analysis. **B, C, D, E** Different models to construct key metabolic pathways (**B** decision tree, **C** random forest, **D, E** SHAP model)

Using the method described by Wu et al., we identified gene sets involved in 46 tumor metabolic pathways in the KEGG database. By GSVA and Limma differential analyses, we identified 12 pathways with *t value* ≥ 1.0 and B-H corrected *p value* < 0.05, including PYRIMIDINE_METABOLISM, PURINE_METABOLISM, and GLYOXYL ATE_AND_DICARBOXYL ATE_METABOLISM. We also identified 25 pathways with *t valuet* ≤ -1.0 and B-H corrected *p value* <0.05, including GLYCOSPHINGOLIPID_BIOSYNTHESIS_LACTO_AND_NEOLACTO_SERIES, ARGININE_AND_PROLINE_METABOLISM, and GLYCOSAMINOGLYCAN_DEGRADATION (Fig. 3A).

### Developing a thymoma machine learning classifier by analyzing distinct metabolic pathways

Differential analysis revealed 37 differential metabolic pathways important for thymoma progression.

The linear regression analysis revealed that there were significant variations in GSAV scores between Cluster1 and Cluster3 across 37 pathways. The pathway variables showed a linear correlation (precision-recall curves: sensitivity and specificity AUC values of 0.586 and recall and precision AUC values of 0.593). Decision tree analysis suggested that KEGG_GLYCOSPHINGOLIPID_BIOSYNTHESIS_LACTO_AND_NEOLACTO_SERIES with a GSVA score of < -0.12 and KEGG_GLYCOSPHINGOLIPID _BIOSYNTHESIS_GLOBO_SERIES with a GSVA score of ≥ 0.12 were the most important pathways for the model (Fig. 3B). Random forest analysis showed that the KEGG_GLYCOSPHINGOLIPID_BIOSYNTHESIS_LACTO_AND_NEOLACTO_SERIES, KEGG_ASCORBATE_AND_ALDARATE_METABOLISM, and KEGG_PRIMARY_BILE_ACID_BIOSYNTHESIS pathways had the highest contribution to the identification of thymoma subtypes with poor prognosis (Fig. 3C). The SHAP model showed that the KEGG_GLYCOSPHINGOLIPID_BIOSYNTHESIS_LACTO_AND_NEOLACTO_SERIES, KEGG_PRIMARY_BILE_ACID_BIOSYNTHESIS, KEGG_TAURINE_AND_ HYPOTAURINE_METABOLISM, KEGG_CITRATE_CYCLE_TCA_CYCLE, and KEGG_ASCORBATE_AND_ALDARATE_METABOLISM pathways were of utmost importance for the model (Fig. 3D). Hence, these algorithms recognized distinct crucial routes, with the decision tree, random forest, and SHAP models concurring that KEGG_GLYCOSPHINGOLIPID_BIOSYNTHESIS_LACTO_AND_NEOLACTO_SERIES held utmost significance among the metabolic pathways. Additionally, we assessed the SHAP-value of this pathway and observed that its GSVA score greatly impacted the classifier output (Fig. 3E).

### Impact of the core pathway on the long-term survival of patients with thymoma

**Figure 4:**
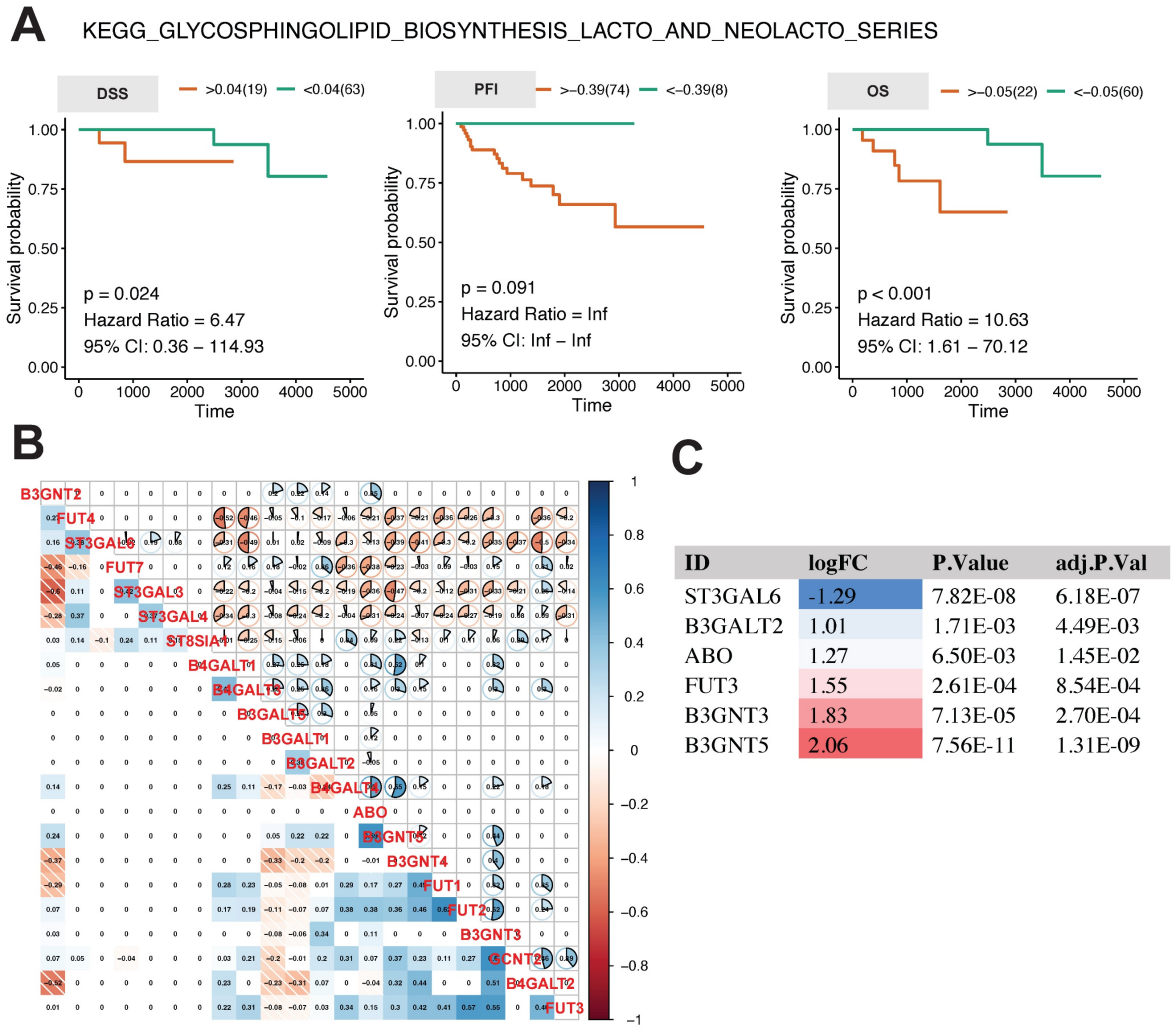
Relationship between core metabolic pathways and long-term survival in thymoma patients. **A** K-M survival analysis of glycosphingolipid biosynthesis lacto and neolacto series. **B, C** Pearson correlation analysis and differential expression analysis of genes in glycosphingolipid biosynthesis lacto and neolacto series.

The analysis above revealed that the glycosphingolipid biosynthesis lacto and neolacto series is the central regulatory pathway linked to the progression of thymoma. In order to examine the influence of this pathway on the future outlook of thymoma patients, we conducted K-M survival analysis using the GSVA score of this pathway. The results in Fig. 4A show that the higher the GSVA score of this pathway was, the worse the prognosis of the patients was, as indicated by DSS (*p* = 0.024, hazard ratio = 6.47, 95% CI: 0.36-114.93), OS (*p* <0.001, hazard ratio = 10.63, 95% CI: 1.61-70.12), and, to a lesser extent, PFI (*p* = 0.091, hazard ratio = Inf, 95% CI: Inf-Inf).

In order to comprehend the expression and interaction of genes within this pathway, we conducted a Pearson correlation analysis on the expression levels of 22 genes involved in this pathway. In general, there was a correlation among the genes, with strong correlations observed between FUT7, ST3GAL3, and ST3GAL4, as well as between B3GNT4 and FUT1 (Fig. 4B). In tumor tissues, the expression of ST3GAL6 was found to be notably reduced, whereas the expression levels of B3GALT2, ABO FUT3, B3GNT3, and B3GNT5 were observed to be significantly elevated (Fig. 4C).

### Metabolomic profiling analysis of thymomas

**Figure 5:**
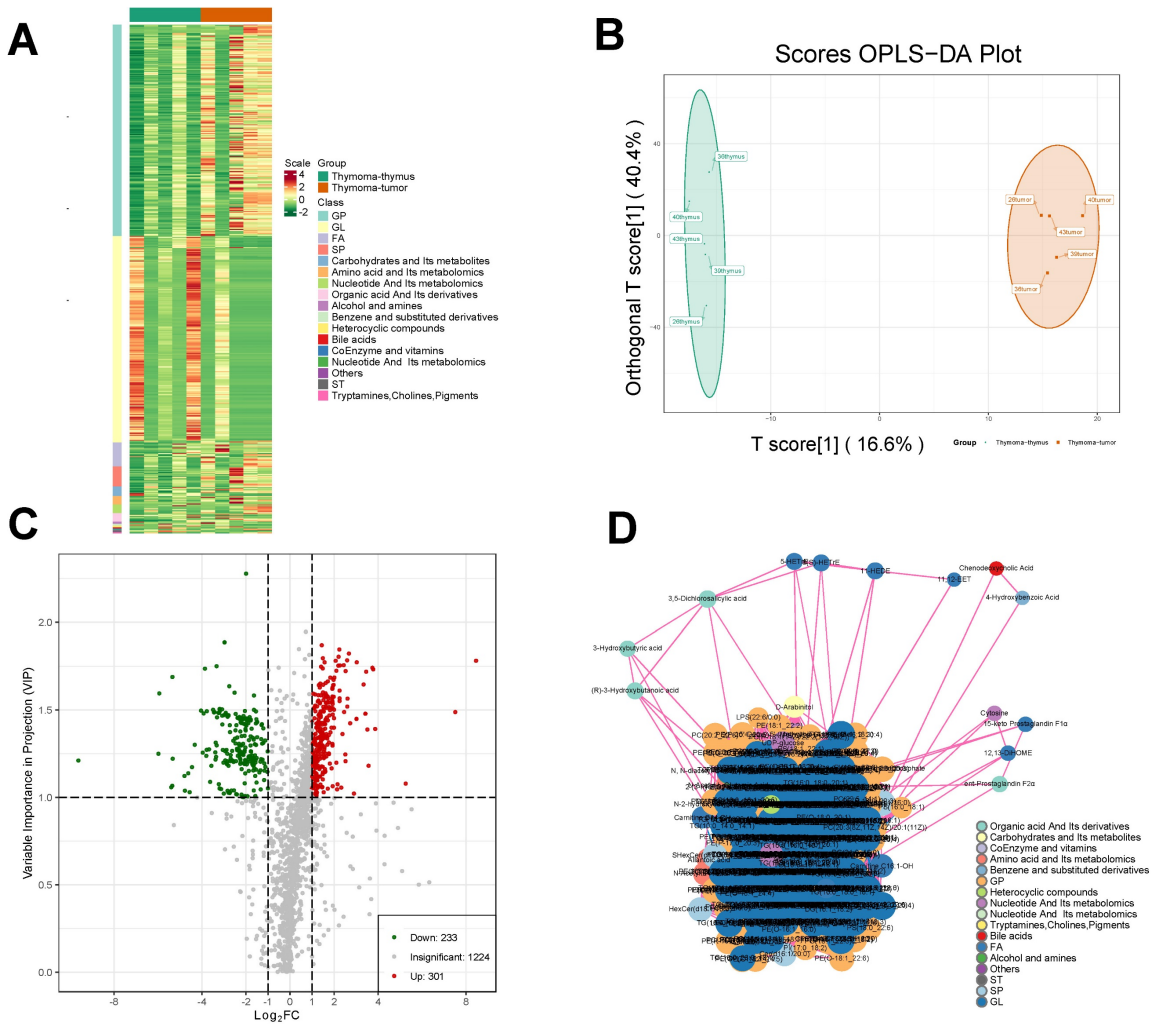
Metabolomic analysis of thymoma samples. **A** Correlation analysis of differential metabolites in thymoma tissue samples and para-tumor tissue samples. **B** The OPLS-DA plot. **C** Screening of differential metabolites D. Interaction diagrams between different metabolites.

Widely targeted metabolomic profiling detected a grand total of 1758 metabolites from the 10 samples. Hierarchical cluster analysis suggested that the distributions of different types of metabolites had certain heterogeneity among groups (Fig. 5A). Unsupervised principal component analysis (PCA) revealed significant metabolic heterogeneity among groups and minimal variation within groups (Fig. 5B).

The coefficient of variation (CV) is the ratio of the standard deviation to the mean of the original data, reflecting the degree of data dispersion. The proportion of substances with CV < 0.3 in the quality control samples was higher than 85%, which was higher than the standard value of 75%, indicating that the experimental data were very stable.

The *Q*^2^ of the OPLS-DA model was 0.542, higher than the acceptable limit of 0.5 for an effective model proposed by Thévenot et al. By the permutation test, there were 93 randomized models with a superior interpretation rate for the matrix to this OPLS-DA model (*P* < 0.05). A total of 534 differential metabolites were selected by VIP, including 233 downregulated metabolites and 301 upregulated metabolites (Fig. 5C). A significant interaction map among different metabolites was detected, as shown in Fig. 5D.

### Functional enrichment analysis of differential metabolites

**Figure 6:**
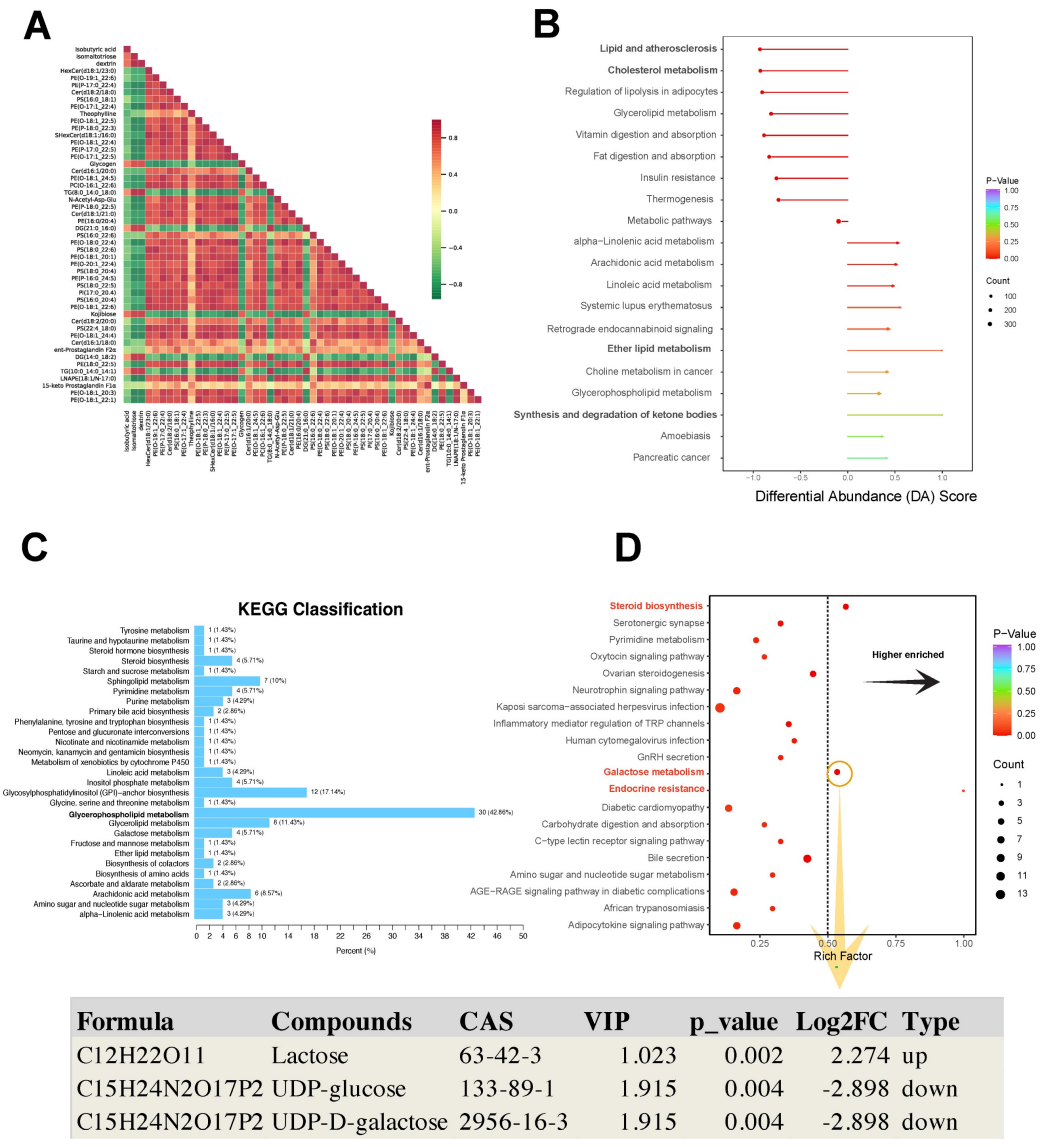
KEGG analysis of differential metabolites. **A** Pearson correlation analysis of differential metabolites. **B** KEGG pathway-based differential abundance (DA) scoring. **C, D** Screening of significantly enriched pathways.

The Pearson analysis showed that different differential metabolites had a synergistic or incompatible relationship (i.e., metabolic proximity), suggesting mutual regulation of differential metabolites (Fig. 6A). The differential abundance (DA) score is a numerical assessment of alterations in metabolism based on KEGG pathways, indicating the average and overall modifications in all metabolites within a particular pathway. The formula for the DA score was DA score = (the number of upregulated differential metabolites in the pathway - the number of downregulated differential metabolites in the pathway)/the number of all metabolites annotated to the pathway. Lipid and atherosclerosis, cholesterol metabolism, and regulation of lipolysis in adipocytes showed a decreased expression trend in the pathological progression process of thymoma. Ether lipid metabolism, and synthesis and degradation of ketone bodies showed an increased expression trend (Fig. 6B).

In the KEGG pathway map related to metabolism, 30 DEMs were enriched in the glycerophospholipid metabolism pathway (Fig. 6C). Furthermore, the galactose metabolism, steroid biosynthesis, and endocrine resistance pathways exhibited significant enrichment with the richness factor > 0.5 (Fig. 6D). In this plot, the compounds lactose (VIP = 1.023, Log2FC = 2.274, *p* value = 0.002), UDP-glucose (VIP = 1.915, Log2FC = -2.898, *p* value = 0.004), and UDP-D-galactose (VIP=1.915, Log2FC = -2.898, *p* value = 0.004) were identified (Fig. 6D).

### Hub regulator detection

**Figure 7:**
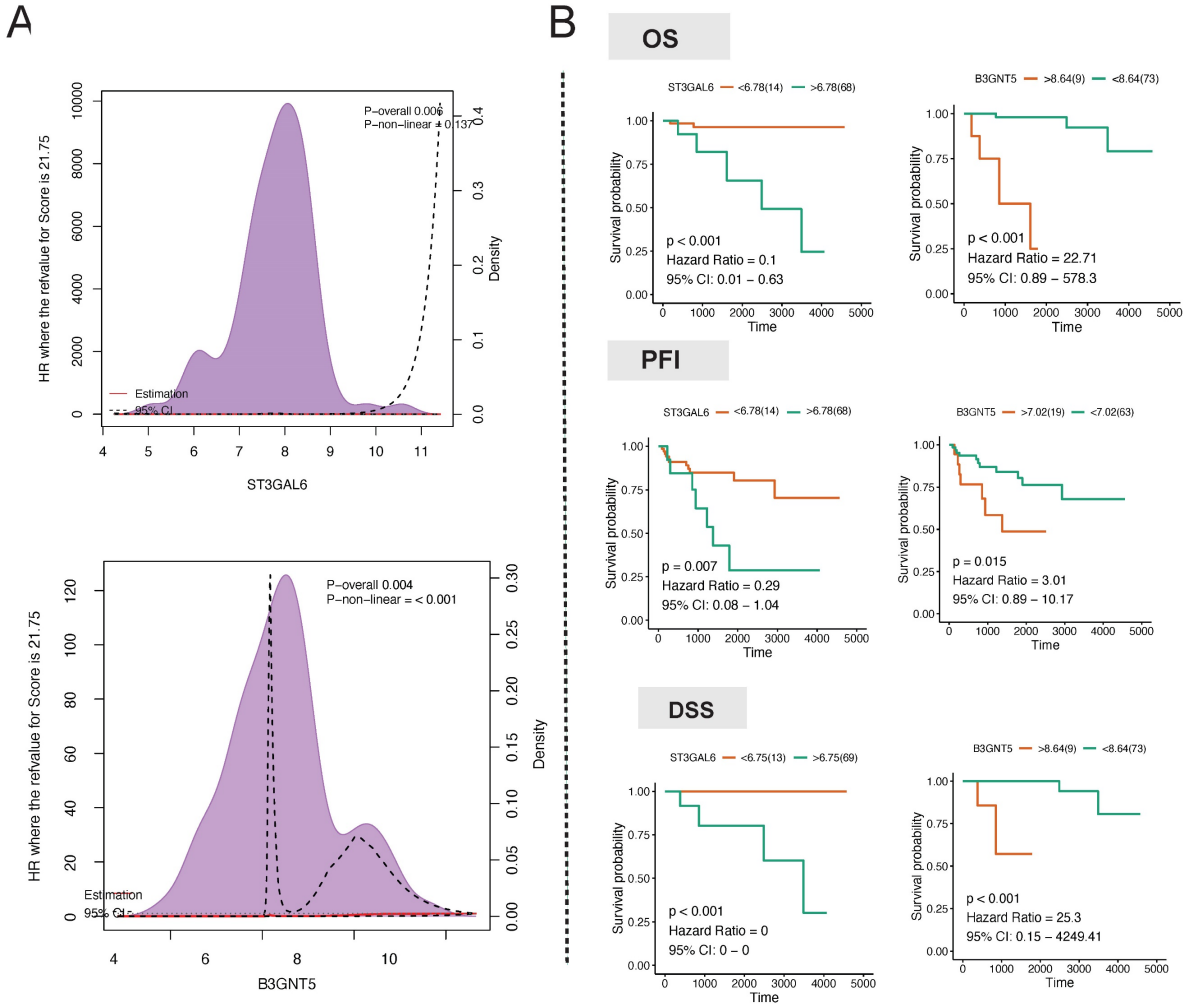
Hub regulator detection. **A** Association analysis of hub gene expression and survival. **B** Long-term survival analysis of hub gene expression in patients with thymoma

In order to mitigate the impact of nonlinear gene expression on survival analysis using transcriptome profiling, we employed the restricted cubic spline model to assess the non-linear impacts of the continuous expression of the target genes ST3GAL6, B3GALT2, ABO, FUT3, B3GNT3, and B3GNT5 on survival. ABO (*p* for combined association = 0.007, *p* for non-linear association = 0.082) and B3GNT5 (*p* for combined association = 0.004, *p* for non-linear association ≤ 0.001) had a notable influence on the overall survival (OS) of thymoma patients (Fig. 7A). The linear continuous expression of ABO and B3GNT5 showed significant impacts on OS (ABO: *p* = 0.056, hazard ratio = 0.26, 95% CI: 0.04-1.93; B3GNT5: *p* < 0.001, hazard ratio = 22.71, 95% CI: 0.89-578.3), PFI (ABO: *p* = 0.058, hazard ratio = 0.38, 95% CI: 0.1-1.53; B3GNT5: *p* = 0.015, hazard ratio = 3.01, 95% CI: 0.89-10.17), and DSS (ABO: *p* = 0.003, hazard ratio = 0.07, 95% CI: 0.01-0.94; B3GNT5: *p* < 0.001, hazard ratio = 25.3, 95% CI: 0.15-4249.41) (Fig. 7B).

### High B3GNT5 expression associated with worse disease-free survival

**Figure 8:**
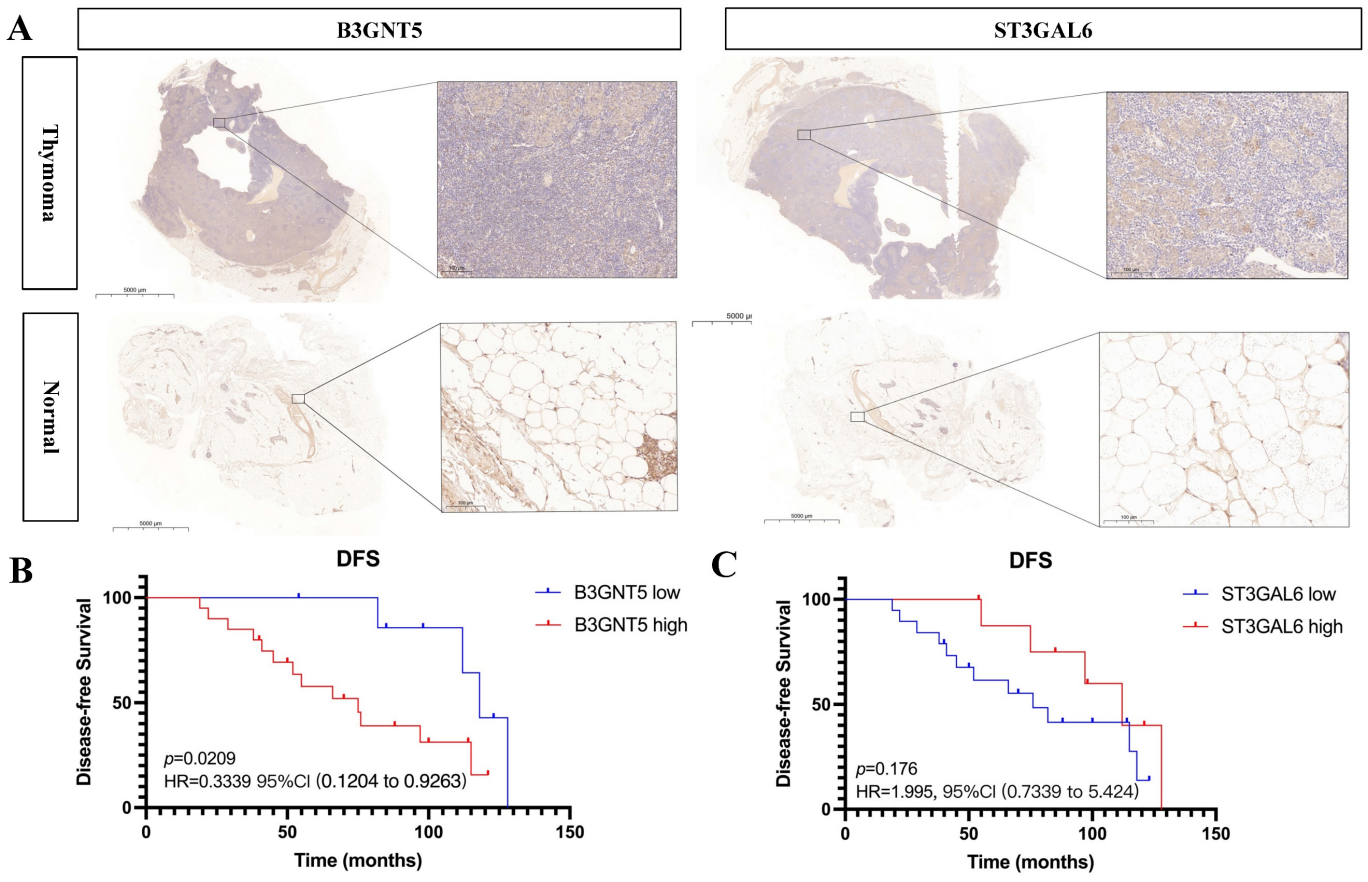
B3GNT5 and ST3GAL6 are upregulated in thymoma tissues and B3GNT5 expression associates with disease-free survival.

Expression of B3GNT5 and ST3GAL6 was upregulated in thymoma tissues than adjacent normal thymic tissues (*p* < 0.05) (Fig. 8). Furthermore, the clinical data showed that high expression level of B3GNT5 in thymoma was associated with worse disease-free survival (*p* < 0.05, HR = 0.3339, 95%CI 0.1204-0.9263), while ST3GAL6 expression didn't influence DFS (*p* > 0.05, HR = 1.995, 95%CI 0.7339-5.424) (Fig. 8).

## Discussion

Due to the marked heterogeneity of thymoma, even with the same histological diagnosis, the prognosis of patients tends to vary significantly. The conversion of healthy cells to cancerous cells is accompanied by various biological characteristics with metabolic reprogramming being the most notable, including glycolysis, glutamate-dependent anabolism, and lipid synthesis 36-38. Hence, exploring novel subtypes of tumors, especially regards to their metabolism, is an effective way to study the heterogeneity of thymoma, thereby providing insights for clinicians to make more accurate clinical assessments 39, 40. The aim of this study was to identify a valuable metabolic gene signature for thymoma by examining the distinct metabolic genes in the TCGA database.

Through investigation, we conducted consensus clustering on thymoma individuals within the database by utilizing genes associated with metabolism. By employing advanced bioinformatics analysis, we successfully pinpointed the crucial involvement of lipid metabolism in thymoma. Organelles contain lipids that serve as essential nutrients for normal cell growth and also function as components of cell membranes 41. Research showed that the alteration of lipid metabolism is vital in membrane synthesis, energy production, and signal transduction during cancer cell progression. Tumor cells rely on lipid metabolism to fuel their energy needs, support cell growth, produce signaling molecules, and prioritize lipid synthesis for rapid proliferation 42-44. The alteration of lipid metabolism is strongly associated with worse tumor prognosis, evidenced in different types of tumors including pancreatic cancer, breast cancer, and non-small cell lung cancer 45-47. Elevated lipid levels can enhance the metastatic potential of cancer cells and contribute to drug resistance. Adjust the sentence structure, delete unnecessary words, and use synonyms to maintain the meaning 48, 49.

Creation of a machine learning classifier helped us to find glycosphingolipid biosynthesis, particularly the lacto and neolacto series, played an important role in thymoma progression. This classification model helped us to identify two key enzymes, namely B3GNT5 and ST3GAL6, that play crucial roles in this process 50-53. Glycosphingolipids (GSLs) are highly varied and plentiful glycolipids that exist on the outer layer of the cell membrane in various living organisms. They are a crucial part of the lipid composition of the plasma membrane in most eukaryotic cells. Their involvement includes processes of cell‒cell recognition and regulation of signals through the regulation of membrane microdomains and proteins associated with the membrane 54, 55. The glycan composition present on the cell surface is a major determinant of the structural and functional classification of GSL, and alterations in GSL glycosylation are associated with stem cell differentiation and contribute to a variety of cancer processes, such as persistent tumor cell proliferation, promotion of tumor cell metastasis 56, 57. The core glycans of GSLs are categorized into three primary groups: the ganglio-series, globo-series, and lacto/neolacto series. Glycosphingolipids from the lacto/neolacto series function as carriers or essential constituents of numerous glycan antigens, which have demonstrated significant involvement in various types of cancer 58. B3GNT5, an essential enzyme involved in the production of lactate and the glycosphingolipids of the lactate series, is crucial in the development of certain cancerous conditions like ovarian cancer and glioblastoma 59, 60. A recent study showed enhanced enzymatic function of B3GNT5 causes the buildup of a fresh lactate sequence of glycosphingolipids in cancer cells, impeding immune monitoring 9. ST3GAL6 belongs to sialyltransferase family, involving in synthesis of glycolipid substrates, abnormalities of which are associated with cancer development, cell adhesion, invasion and metastasis 61. ST3GAL6 is excessively expressed in different types of cancers. For instance, it stimulates the growth and invasion of hepatocellular carcinoma and colon cancer cells through the PI3K/AKT signaling pathway. Additionally, its overexpression enhances the ability of gastric cancer cells to inhibit the resistance of the Met tyrosine kinase receptor to crizotinib treatment 62. Nevertheless, the results from the TCGA repository indicate a significant correlation between increased B3GNT5 levels and unfavorable prognosis, whereas ST3GAL6 acts as a gene that provides protection. This result was validated in our tissue samples but remains to be further elucidated.

To summarize, our examination revealed two molecular categories linked to metabolism in thymoma, and we investigated the metabolic routes and crucial genes implicated in thymoma.The results of our study provide new insights into the classification of thymoma and emphasize its involvement in the heterogeneity of tumors related to metabolism. However, further validation requires larger sample sizes.

## Conclusions

This study revealed a strong correlation between the glycosphingolipid biosynthesis pathway and thymoma. B3GNT5 can be used as potential biomarkers to predict better prognosis of thymoma patients.
